# The role of Vitamin D in immuno-inflammatory responses in Ankylosing Spondylitis patients with and without Acute Anterior Uveitis

**Published:** 2016

**Authors:** TC Mitulescu, C Stavaru, LM Voinea, LM Banica, C Matache, D Predeteanu

**Affiliations:** * Department of Ophthalmology, University Emergency Hospital, Bucharest, Romania; ** Cellular and Molecular Immunity Laboratory, Cantacuzino National Institute for Research, Bucharest, Romania; *** ”Carol Davila” University of Medicine and Pharmacy, Bucharest, Romania; **** Department of Rheumatology, “Sf. Maria” Clinical Hospital, Bucharest, Romania

**Keywords:** Ankylosing Spondylitis, Vitamin D, LL-37, IL-8, SAA

## Abstract

**Hypothesis:**Abnormal Vitamin D (Vit D) level could have consequences on the immuno-inflammatory processes in Ankylosing Spondylitis (AS).

**Aim:**The purpose of this study was to analyze the role of Vitamin D in the interplay between immune and inflammation effectors in AS associated-Acute Anterior Uveitis (AAU).

**Methods and Results:**25-hydroxyvitamin D (Vit D), LL-37 peptide, IL-8 and Serum Amyloid A (SAA) were identified and quantified in the serum/ plasma of thirty-four AS patients [eleven AS patients presenting AAU (AAU AS patients) and twenty-three AS patients without AAU (wAAU AS patients)] and eighteen healthy individuals (Control) using enzyme-linked immunosorbent assay. Acute-phase SAA level was significantly higher in AS patients compared to Controls. Contrary with wAAU AS patients, significantly elevated levels of IL-8, and diminished levels of Vit D characterized AAU AS patients. Regarding LL-37, its level decreased concomitantly with the level of Vit D. When AS patients were subgrouped based on AAU presence or on Vit D level, important associations between immuno-inflammatory assessed markers and AS features were noticed. Generally, Vit D levels were associated indirectly with leukocytes/ neutrophils number or with ESR, CRP, and Fibrinogen levels. The levels of SAA and IL-8 associated directly with AAU or with AAU relapses, especially in AS patients with Vit D insufficiency, while SAA associated directly with infection/ inflammatory markers and with disease activity indexes or with the degree of functional limitation.

**Discussion:**Altered levels of Vit D affect the balance between LL-37, IL-8 and SAA, suggesting an association with AAU, an extra-articular manifestation of AS.

**Abbreviations:**Vit D = Vitamin D, AS = Ankylosing Spondylitis, AAU = Acute Anterior Uveitis, AAU AS = AS patients with AAU, wAAU AS = AS patients without AAU, SSZ = Sulphasalazine, Leu = Leukocytes, Neu = Neutrophils.

## Introduction

Acute Anterior Uveitis (AAU) belongs to the clinical picture of Ankylosing Spondylitis (AS), the prototype of spondyloarthritis. Though the disease etiology is still incompletely elucidated, both genetic and environmental factors, namely pathogens, were involved [**1**,**2**,**3**]. About 50% of the patients with AAU were positive for HLA-B27, a class I major histocompatibility complex molecule. These patients were a distinct clinical phenotype frequently characterized by ocular inflammation associated with systemic inflammatory manifestations. AAU was considered the prototype of immune-mediated ocular inflammation. Infections with Chlamydia trachomatis and gram-negative bacteria species (Salmonella, Shigella, Campylobacter, Klebsiella, and Yersinia) were involved in the pathogenesis of HLA-B27-associated AAU [**4**]. The precise pathogenic mechanism remains unclear. Until now, it was assumed that molecular mimicry, pro-inflammatory cytokines, and Toll-like receptors (TLRs) trigger host innate immune response to microbial components [**2**,**5**]. Thus, bacterial lipopolysaccharide, by TLR-4, stimulates the iris pigment epithelial cells and activates the secretion of pro-inflammatory chemokines (including IL-8) [**6**]. When antigen-presenting cells from perivascular areas of the uvea are targeted by TLR-4, they are recruited into the uvea. This process is mediated by the stimulation of adhesion molecules expression and chemokines secretion, including IL-8 [7]. In AS patients, monocytes overexpress some TLRs and monocytes activation by TLRs leading to an exacerbated secretion of cytokines such as, TNF and finally to inflammation [**8**,**9**]. Additionally, the increased production of TNF maintains TLRs overexpression.

Inflammation or infections of the ocular surface were associated with a weak expression of antimicrobial peptides (AMPs). AMPs, among which human cathelicidin LL-37 peptide, are microbicidal cationic and cellular signaling peptides for the host’s response to pathogens [**10**]. The study of the relationship between LL-37 expression, cell proliferation and cytokine production in human corneal epithelial cells, revealed that the increase of LL-37 expression after bacterial attack contributed to cytokines production and bacteria destruction without increasing cellular proliferation, thus suggesting a multifunctional role for LL-37 [**11**]. Moreover, in a murine model of endotoxin-induced uveitis it has been suggested that cathelicidin hCAP18 (109-135), may be a promising agent for the treatment of ocular inflammation [**12**]. Decreased expression of AMPs in humans has been associated with an exacerbation of pro-inflammatory cytokines production, including IL-1, IL-6, IL-8, IFN-?, and TNF-a [**11**]. In addition, infections or injuries cause the release into circulation of a large amount of Serum Amyloid A (SAA) [**13**]. SAA is a potent chemoattractant for neutrophils (Neu) and monocytes and it stimulates Neu for IL-8 production [**14**]. Once secreted excessively, IL-8 leads to adverse effects in the body such as inflammation and tissue destructions [**15**]. In contrast, human cathelicidin LL-37 peptide, acting on formyl peptide receptor like-1 (FPRL-1) has the ability to inhibit IL-8 secretion and Neu migration induced by SAA, revealing anti-inflammatory properties of AMPs [**16**].

The active form of Vitamin D, 1,25-dihydroxyvitamin D3, increases the expression of AMPs, mainly LL-37, in many different types of cells [**17**]. A Vitamin D response element was found on the promoter of the genes encoding AMPs [**18**]. In patients with AS, the data regarding Vitamin D status are contradictory [**19**,**20**,**21**,**22**]. Jung et al. [**23**], demonstrated an association between the polymorphism of Vitamin D binding protein (DBP) gene encoding DPB protein transporting Vitamin D and its metabolites and the development of peripheral arthritis and uveitis in Korean patients with AS. Furthermore, Steinwender et al. [**24**] have suggested that in HLA-B27-associated uveitis, an important role may be played by the polymorphism of CYP27B1 gene encoding 25-hydroxyvitamin D-1 alpha hydroxylase (CYP27B1) involved in Vitamin D metabolism. 

Based on this data, the aim of this study was to evaluate some of the key elements of pro- and anti-inflammatory processes (IL-8, SAA, and LL-37, respectively) and their relationship with Vitamin D level and AS features. Our results showed that Vitamin D, LL-37, SAA and IL-8 interplay are involved in AS pathogenesis, being associated with some conventional characteristics for AS. 

## Materials and methods Patients and Control

Fifty-two subjects were enrolled in this study: thirty-four AS patients and eighteen apparently healthy individuals constituting the Control group. During the study, eleven AS patients presented AAU (AAU AS) while twenty-two AS patients were without AAU (wAAU AS). Selection and characterization of AS patients was performed based on modified New York criteria [**25**]. Between March 2014 and April 2015, subjects were enrolled in the study. The disease activity and the degree of functional limitation were also determined for each patient according to Bath Ankylosing Spondylitis Disease Activity Index (BASDAI) [**26**] and Ankylosing Spondylitis Disease Activity Score (ADSAS) [**27**] and, Bath Ankylosing Spondylitis Functional Index (BASFI) [**28**], respectively. When the study design was developed, neither AS patients nor Control individuals were treated with Vitamin D. AS patients received only non-biological treatment consisting in nonsteroidal anti-inflammatory drugs (NSAIDs) and/ or Sulphasalazine (SSZ) at the time the biological samples were taken. The study was approved by the Ethics Committee of involved institutions according to the World Medical Association’s Declaration of Helsinki, revised in 2000, Edinburgh. An informed consent for all investigations on human individuals was obtained and patient anonymity was preserved. **Table 1** shows the demographic, clinical and paraclinical characterization of AS patients and Controls.

**Table 1:**
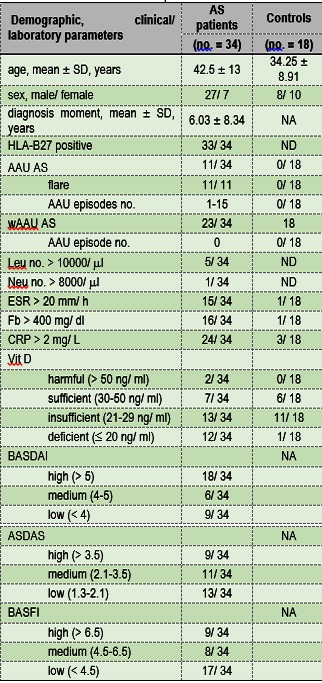
**Table 1.**Characterization of AS patients and Controls

no. = number, NA = Not Applicable, ND = Not Determined

### Biological specimen

Plasmas from peripheral blood of AS patients and Control individuals were obtained according to the manufacturer’s instructions for LL-37 Enzyme-Linked Immunosorbent Assay (ELISA) kit, Hycult Biotech, Uden, Netherlands. Once obtained, the plasma samples were stored at -80°C until LL-37, IL-8 and SAA were assessed. The sera resulted by clotting and blood centrifugation at 420 g were stored at +4°C and processed for 25-hydroxyvitamin D (Vit D) levels within 48 h. 

### Quantitative assessment of Vit D level in AS patient and Control sera

Vit D level was quantified in AS patients and Control sera by using EUROIMMUN 25-OH-Vitamin D ELISA kit (Luebeck, Germany) according to the manufacturer’s protocol. Vit D status in the body was characterized as sufficient (Vit D = 30-50 ng/ ml), insufficient (Vit D = 21-29 ng/ ml) or deficient (Vit D < 20 ng/ ml) [**29**,**30**]. 

### Quantitative assessment of circulating levels of LL-37, IL-8, and SAA

To evaluate the levels of LL-37, IL-8 and SAA in the plasma samples of AS patients and Control individuals, commercial ELISA kits for human LL-37 and for human IL-8 and SAA (MyBioSource, San Diego, USA) were used. The assays were performed according to the manufacturer’s protocols. Plasma samples were diluted 1/ 500 and 1/ 50 to quantify LL-37 and SAA levels, respectively, or were used undiluted for IL-8 level quantification. The concentrations of LL-37 and SAA were expressed in ng/ ml while IL-8 concentration in pg/ ml.

### Statistical analysis

Statistical analyses of the data and figures design were performed by using GraphPad Prism 6 software (http://www.graphpad.com/demos/). Mann-Whitney U test was used to compare the mean values between groups/ subgroups. Spearman’s rank nonparametric correlation test (two-tailed) was used to evaluate the relationship between assessed variables. The statistical significance was set for a P value < 0.050.

## Results

**Vit D, LL-37, IL-8, and SAA levels under normal and pathologic conditions**

Sera or plasmas isolated from thirty-four AS patients and eighteen apparently healthy donors (Control) were analyzed for the levels of Vit D, LL-37, IL-8, and SAA by using ELISA. As it can be seen in **Table 2**, except for the SAA, the other immuno-inflammatory markers appeared to be comparable between AS patient and Control groups. However, this analysis highlighted the following aspects: only six of eighteen Control individuals have had a sufficient level of Vit D and two of thirty-four AS patients with AAU have had a harmful level of Vit D (> 50 ng/ ml). Therefore, to avoid misinterpretations, for subsequent statistical analyses, we did not use the comparison between AS patient subgroups and Control and, the two AAU AS patients with harmful level of Vit D were removed from AS patients group.

**Table 2.** Vit D, LL-37, IL-8 and SAA levels in AS patients and Control. Using commercial ELISA kits, the levels of Vit D, LL-37, IL-8, and SAA were quantified in serum/ plasma of thirty-two AS patients and eighteen Controls. Mean values, standard deviations (SD), median values, and sum of ranks for each group of individuals and each immune/ inflammatory assessed marker were calculated. The differences between AS patient and Control groups were established based on Mann-Whitney U test (p<0.050).

**Table. 2:**
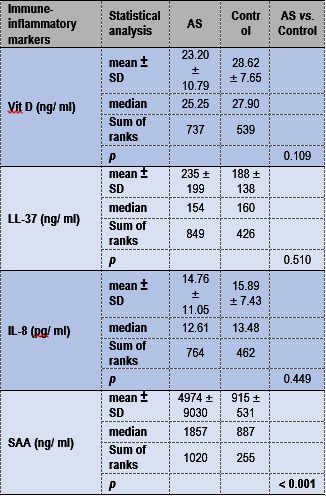
**Table 2**Immuno-inflammatory assessed markers that differentiated between AAU AS and wAAU AS patients

**Fig. 1:**
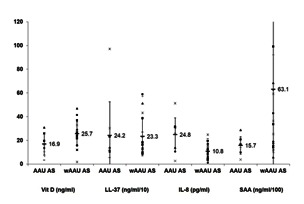
**Fig. 1**Vit D, LL-37, IL-8, and SAA levels in AS patients. Using commercial ELISA kits the levels of Vit D, LL-37, IL-8, and SAA were quantified in serum/ plasma of thirty-two AS patients. AS patients were distributed into subgroups based on AAU presence and the results for Vit D, LL-37, IL-8, and SAA levels are presented as dot plots. The concentrations of LL-37 and SAA in plasma samples were divided by 10 and 100, respectively. Mean values ± standard deviations (SD), calculated for each immuno-inflammatory assessed markers and each subgroup of AS patients, are included

The levels of Vit D, LL-37, IL-8, and SAA in the sera/ plasmas isolated from AAU AS and wAAU AS patients were quantified by using ELISA. The results obtained on these subgroups of AS patients are presented in **Fig. 1.** As it can be seen, AAU AS patients had lower levels of Vit D and SAA and higher levels of IL-8 levels compared to wAAU AS patients. However, only IL-8 levels significantly discriminated between AAU AS and wAAU AS patient groups (IL-8 median 26 pg/ ml, sum of ranks 214 in AAU AS patients and IL-8 median 10.13 pg/ ml, sum of ranks 314 in wAAU AS patients, p = 0.006 by Mann-Whitney U test). Nevertheless, Vit D level tends to discriminate between AAU AS and wAAU AS patient groups (Vit D median 16.50 ng/ ml, sum of ranks 102 for AAU AS patients and Vit D median 26.40 ng/ ml, sum of ranks 426 in wAAU AS patients, p = 0.053 by Mann-Whitney U test). Probably, due to a large dispersion of the data, SAA could not statistically discriminate between AAU AS and wAAU AS patients (p = 0.094). Regarding AMPs, the levels of LL-37 seemed to be comparable between AAU AS and wAAU AS patient subgroups. 

### SAA differentiates between AS patients with altered Vit D level

**Fig. 2:**
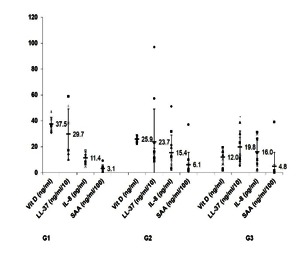
**Fig. 2**The distribution of immuno-inflammatory assessed markers depending on Vit D status in AS patients. AS patients were divided into subgroups based on serum Vit D level as it follows: AS patients with Vit D sufficiency (G1), AS patients with Vit D insufficiency (G2) and AS patients with Vit D deficiency (G3). The results for Vit D, LL-37, IL-8, and SAA levels quantified by ELISA are presented as dot plots. The concentrations of LL-37 and SAA in plasma samples were divided by 10 and 100, respectively. Mean values ± standard deviations (SD), calculated for each immuno-inflammatory assessed markers and each subgroup of AS patients, are included

To determine the link between Vit D and effectors of innate immunity and of inflammation in AS, AS patients were distributed depending on their serum Vit D level into AS patients with Vit D sufficiency, insufficiency and deficiency. The levels of immuno-inflammatory assessed markers in these AS patient subgroups are presented in **Fig. 2**. As can be seen in **Fig. 2**, while the LL-37 level decreased with the level of Vit D, IL-8 levels increased. However, except for Vit D level, SAA level was the only one marker that differentiated significantly between AS patients with Vit D insufficiency (SAA median 2535 ng/ ml, sum of ranks 211) and AS patients with Vit D deficiency (SAA median 1579 ng/ ml, sum of ranks 114) (p = 0.024).

### Relationship between Vit D, IL-8 and SAA and clinical AS features

The role of Vit D, LL-37, IL-8, and SAA in AS pathogenesis was evaluated based on statistically significant correlations between these immuno-inflammatory markers and clinical/ paraclinical AS features. These associations were tested by using Spearman’s rank correlation. On the entire group of AS patients, some statistically significant correlations were found. As it can be seen in **Table 3**, serum Vit D level correlated inversely with Leukocytes (Leu) number and with AAU presence. The level of IL-8 correlated directly both with AAU presence and the number of AAU episodes. Regarding the SAA level, positive correlations with Leu and Neu number or with the level of ESR, CRP, Fb were identified (**Table 3**). 

**Table 3.**
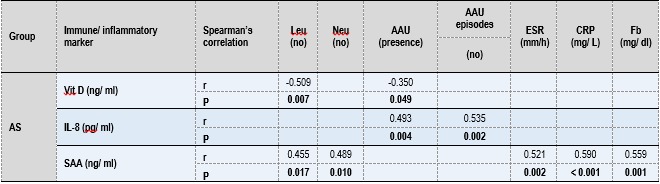
**Table 3**Relationship between immuno-inflammatory assessed markers and clinical/ paraclinical AS features. The level of each immune/ inflammatory assessed marker was correlated with each established clinical and laboratory markers for AS by using Spearman’s rank nonparametric correlation test (two-tailed). Insignificant results (P >0.050) were not included

When the same analysis was applied separately to AAU AS and wAAU AS patient subgroups, some statistically significant correlations were also identified (**Table 4**). In AAU AS patients, SAA levels correlated directly with the number of AAU episodes and with BASDAI and BASFI. In wAAU AS patients, SAA levels correlated directly both with Leu and Neu number and with the values of ESR and CRP. Only in wAAU AS patients, Vit D level correlated inversely with Leu and Neu number as well as with Fb level (**Table 4**). 

**Table 4.**
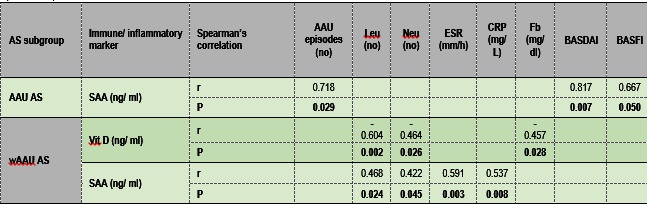
**Table 4**Relationship between Vit D and SAA and clinical/paraclinical AS features in AAU AS and wAAU AS patients. For each subgroup of AS patients (AAU AS and wAAU AS), the levels of immuno-inflammatory assessed markers were correlated with each established clinical and laboratory markers using Spearman’s rank nonparametric correlation test (two-tailed). Only significant results (P <0.050) were included.

To deepen further the role of Vit D in the regulation of innate immunity and inflammation in AS, immuno-inflammatory assessed markers and clinical/ paraclinical AS features were correlated in AS patient subgroups with different Vit D levels (**Table 5**). Thus, in AS patients with Vit D insufficiency, inversely correlations between Vit D and SAA levels or between Vit D level and Neu number were identified. Moreover, in the same AS patients subgroup, IL-8 level correlated directly with AAU recurrence while the SAA level correlated directly with the Neu number. Both IL-8 and SAA levels correlated directly with Neu number in AS patients with deficiency of Vit D. In addition, SAA correlated directly with the values of inflammatory markers (ESR, CRP, Fb) and inversely with NSAIDs therapy. 

**Table 5.**
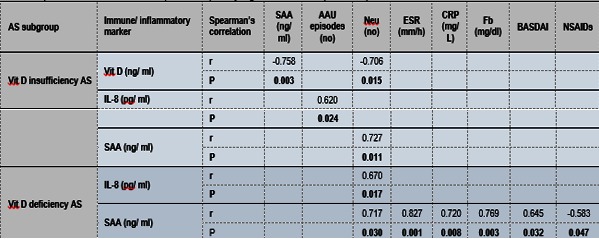
**Table 5**Relationship between Vit D and SAA and clinical/paraclinical AS features in AAU AS and wAAU AS patients. For each subgroup of AS patients (AAU AS and wAAU AS), the levels of immuno-inflammatory assessed markers were correlated with each established clinical and laboratory markers using Spearman’s rank nonparametric correlation test (two-tailed). Only significant results (P <0.050) were included.

## Discussion

In this study, based on previous observations [**19**,**20**,**21**,**22**,**23**,**24**, **31**,**32**,**33**], we tried to simultaneously apprehend the interplay between Vit D, LL-37, IL-8 and SAA in AS patients, focusing on the AS associated-AAU.

Our results showed that, except for SAA, the others immuno-inflammatory experimentally assessed markers were similar in AS patient and Control groups. The Control group presented a substantial proportion of individuals with low levels of Vit D. Recently [**34**], it has been demonstrated that the Vit D level has seasonal variation and fluctuates with age and gender of individuals. In our study, the distribution by gender and age was different among the AS patient and the Control groups. However, we identified no statistically significant correlation between Vit D level and age or gender of individuals in AS patient or Control groups.

In the AS patients group, a negative correlation between Vit D level and Leu number, involved in innate/ adaptive responses, was identified. Furthermore, Vit D levels correlated negatively with the presence of AAU, once again proving the relationship between metabolic alterations of Vit D and AAU development. Vit D levels distinguished AAU AS patients from those wAAU, being significantly lower in AAU AS patients. Based on previous publications, the decrease of Vit D levels in AAU AS patients could be explained by polymorphisms in genes encoding proteins involved in Vit D metabolism or transport [**23**,**24**]. On the other hand, negative correlations between Vit D and Leu or Neu number or Fb level in wAAU AS patients suggested the involvement of Vit D in limiting accompanying events of innate/ adaptive immune or inflammatory processes [**35**,**36**,**37**]. Moreover, in AS patients with insufficient levels of Vit D, Vit D level correlated negatively not only with the Leu number but also with SAA level, emphasizing the regulatory role of Vit D in limiting some of the innate immune and inflammation effectors. Altered levels of Vit D in the body have been shown to decrease the resistance to colonization by various bacteria and led to altered activity/ functions of Neu [**38**,**39**]. The relationship between Vit D and SAA has been previously observed in a group of patients with type 2 diabetes [**40**]. Our results were also in agreement with previous data, showing statistically significant negative correlations between 25-hydroxyvitamin D3 and inflammatory markers in insufficient plus deficient AS patients group [**19**]. It was demonstrated that Vit D deficiency could contribute to a Th1 response [**41**], thus, Vit D deficiency might amplify the inflammatory responses contributing to the disease’s activity strengthening in AS patients.

Since Vit D targets cathelicidin AMPs encoding genes, including human cationic antimicrobial protein-18 (hCAP-18), a molecule considered a promising agent for the treatment of ocular inflammation, we have analyzed LL-37 in AS patients and its association with AAU, taking into consideration the previous observations [**12**,**42**,**43**]. We observed that the lowest LL-37 levels characterized AS patients with insufficiency and deficiency in Vit D, although there were no statistically significant differences between the compared AS patient subgroups.

By its dual activity, LL-37 may act as pro- or anti-inflammatory molecule, stimulating or inhibiting pro-inflammatory cytokines such as IL-8; LL-37 inhibiting in transcriptional and post-transcriptional manner SAA-induced IL-8 production [**11**,**12**,**16**]. Therefore, we evaluated by comparisons the level of IL-8 in AAU AS and wAAU AS patients. The highest levels of IL-8 characterized AAU AS patients, differentiating them from wAAU AS patients. Kramer et al. [**44**] also demonstrated the highest IL-8 levels in patients with active uveitis and a strong association with active disease. Moreover, in the AS patients group, IL-8 correlated directly with AAU presence and with the number of AAU episodes. When Vit D level decreased (AS patients with Vit D insufficiency or deficiency), increasing levels of IL-8 were directly associated with AAU relapses and with Neu number. These associations suggested that as the disease progressed, the inflammation effectors contributed to the extra-articular manifestations of AS, such as AAU. 

SAA was included among the factors produced in the early hours of the acute infectious or inflammatory processes [**45**]. Elevated levels of SAA were identified in AS patients especially in wAAU AS patients, as compared with Control or AAU AS patients, respectively. Furthermore, the SAA level correlated directly with infection/ inflammatory markers (Leu and Neu number, ESR, CRP and Fb) depending on AS features, underlining its inflammatory potential. Previously, it was suggested that SAA and CRP evaluation supported the selection of AS patients and anti-TNF therapy monitoring [**46**]. Interestingly, in AAU AS patients, SAA correlated directly with AAU recurrences, disease activity (BASDAI) and functional limitation degree (BASFI). In AS patients with Vit D deficiency, SAA correlated directly with the disease activity (BASDAI) but also with infection/ inflammatory markers (Neu number, ESR, CRP and Fb). Jung et al. [**33**] previously showed positive correlations between SAA levels and BASDAI, ESR and CRP in AS patients. Taken together, these findings suggested that SAA could be a valuable indicator for the acute disease but also for AAU recurrence. Moreover, NSDAIDs therapy was able to downregulate SAA level in AS patients with Vit D deficiency, proving its efficiency in terms of acute inflammation limitation.

By this study, we pointed out that the AS patients, in particular those with AAU, are characterized by the deterioration of the control mechanisms of immune and inflammatory processes. At least partially, this deterioration seemed to be a consequence of Vit D metabolism alteration and of the overproduction of IL-8. Although insufficiently established, low LL-37 production cannot counter-balance the release of pro-inflammatory mediators, such as IL-8. Until now, there have been no studies regarding the connection between these mediators of immune and inflammatory processes in AS in which Vit D could be a regulatory bridge. Therefore, further studies investigating Vit D, LL-37, IL-8 and SAA levels in AS patients on a larger cohort of subjects are required. Regarding the AS associated-AAU, repeating the measurements once the flare has subsided could be useful to establish the impact of Vit D and the need of Vit D replacement therapy in controlling the pro-inflammatory mediators.

### Acknowledgements

The authors would like to thank their colleagues, Daniela Florescu and Doina Proteasa, from „Cantacuzino” National Institute for Research, Bucharest, Romania, for the technical support.

### Declaration of interest

The authors report no conflict of interest. The authors alone are responsible for the content and writing of the paper.

### Financial support

Ministry of Education and Scientific Research (Romania) (grant number PN09220201) and internal funding supported this work.
